# Bilateral Multivalvular Infective Endocarditis Presenting as a Splenic Infarction and Acute Ischemic Stroke in a Young Immunocompetent Woman

**DOI:** 10.7759/cureus.77942

**Published:** 2025-01-24

**Authors:** Tetyana Okan, Mehrdad Zarghami, Aashish Patel, Suresh Jain, Gagandeep Singh

**Affiliations:** 1 Internal Medicine, Jamaica Hospital Medical Center, New York, USA; 2 Neurocritical Care, Northwell Health, New York, USA; 3 Interventional Cardiology, Jamaica Hospital Medical Center, New York, USA

**Keywords:** bilateral endocarditis, echocardiography for infectious endocarditis, endocarditis in immunocompetent patient, infectious endocarditis, patent foramen ovale (pfo), pfo, right- and left-sided endocarditis, splenic infarction, streptococcus mitis bacteremia, stroke

## Abstract

Bilateral multivalvular infective endocarditis (MIE) involving two or more cardiac valves of both the left and right sides of the heart is an extremely rare disease with a high mortality rate. We present a rare case of left- and right-sided MIE caused by *Streptococcus mitis/oralis* in a 36-year-old immunocompetent woman. The patient, with a past medical history of heart murmur since childhood, presented with left upper quadrant (LUQ) pain, fever, and chills. In the emergency department (ED), the patient's mental status deteriorated. On a physical examination, a holosystolic heart murmur was heard at the apex. The abdomen was tender in the LUQ. Neurological examination showed new right lower and upper extremity weakness. Laboratory results were significant for neutrophilic leukocytosis. The electrocardiogram (EKG) showed the right bundle branch block. Chest computed tomography (CT) of the abdomen and pelvis revealed splenomegaly with a splenic infarct. Magnetic resonance imaging (MRI) of the brain showed acute ischemic infarction of the left middle cerebral artery distribution. The patient underwent a mechanical thrombectomy. A repeat MRI of the brain showed a hemorrhagic conversion; thus, the patient was on hemicrania watch for five days. Transthoracic echocardiography (TTE) revealed left- and right-sided infective endocarditis (IE) with mobile 14 mm and 20 mm vegetations on the mitral and tricuspid valves and a left-to-right shunt through a patent foramen ovale (PFO). Blood culture grew *Streptococcus mitis/oralis*. Six-week antibiotic therapy was initiated. Surgical intervention for infective endocarditis was recommended, and the patient was transferred to the tertiary center for valve replacement surgery. However, the patient refused surgery despite understanding the risks and decided to return to her home country. In conclusion, bilateral multivalvular infective endocarditis is a rare and complex condition with a high burden of complications. This case underscores the importance of early diagnosis, prompt initiation of antimicrobial therapy, and timely surgical intervention to optimize outcomes.

## Introduction

Multivalvular infective endocarditis (MIE) involving two or more cardiac valves occurs in less than 30% of all cases of infective endocarditis (IE), while the incidence of bilateral MIE is only 12% [1]. Infective endocarditis is usually associated with immunocompromised states, intravenous (IV) drug use, and prosthetic valves leading to endothelial injury with subsequent platelets and fibrin deposition at these damaged areas creating a nidus for bacterial adherence and the formation of vegetations. These vegetations, composed of microbes, inflammatory cells, and thrombotic debris, cause local tissue destruction and lead to systemic complications through embolization [2]. The major complications of MIE include congestive heart failure (64%), acute renal failure (50%), embolic events (21%), and splenic abscess/infarcts (21%) [3]. Embolization to the brain with stroke consequences in young patients, including permanent cognitive deficits, epilepsy, and chronic debilitating fatigue, carries an enormous economic burden for the US healthcare system [4].

It is essential to differentiate bilateral endocarditis from nonbacterial thrombotic endocarditis (NBTE), also known as marantic endocarditis. NBTE is associated with a hypercoagulable state, such as malignancy or autoimmune conditions, and features sterile vegetations composed of fibrin and platelets. While marantic endocarditis does not directly cause infection, its vegetations can embolize, mimicking the systemic complications seen in IE. This distinction is critical, as treatment strategies differ significantly, with NBTE primarily requiring anticoagulation rather than antimicrobial therapy [5].

The mortality rate of IE is high, accounting for up to 30% at 30 days with particularly high mortality in critically ill patients, ranging from 29% to 84% [1,6]. We present a rare case of left- and right-sided MIE caused by *Streptococcus mitis/oralis* in a young woman with no known immunocompromised conditions and no history of IV drug use or prosthetic valve who presented with splenic infarct and acute stroke.

## Case presentation

A 36-year-old woman from Ecuador with a past medical history of heart murmur since childhood presented to the emergency department (ED) with worsening left upper quadrant (LUQ) pain, fever, and chills that started one month ago. At the initial presentation, the patient was alert and oriented in person, time, and place and was able to speak and explain her symptoms. The patient denied chest pain, shortness of breath, palpitations, or cough; denied any history of rheumatic heart disease, HIV, diabetes, any surgical history, IV drug use, malignancy, or autoimmune conditions; and was not on any medications at home. In ED, the patient's mental status deteriorated, and the patient became lethargic and unable to communicate. Initial vital signs were as follows: heart rate (HR), 114 beats per minute (bpm); blood pressure (BP), 99/69 mmHg; respiratory rate (RR), 17; temperature, 38.1°C; and oxygen saturation (SpO_2_), 89% with improvement on nasal cannula. Physical examination was remarkable for poor dentition, and a holosystolic heart murmur was best heard at the apex and the left upper sternal border radiating to the left axilla. The abdomen was tender in LUQ but soft and nondistended. No lymphadenopathy or any skin changes were noticed. Laboratory results were significant for neutrophilic leukocytosis, microcytic anemia, mild transaminitis, and elevated ESR and C-reactive protein (CRP); urinalysis was positive for leukocytes, erythrocytes, and bacteriuria with no symptoms of urinary tract infection (Table 1). The initial diagnosis was sepsis due to suspected meningitis with differential diagnoses including stroke and infective endocarditis.

**Table 1:** Laboratory values of the patient. Abnormal values are in bold text. MCV, mean corpuscular volume; MCH, mean corpuscular hemoglobin; MCHC, mean corpuscular hemoglobin concentration; RDW, red cell distribution width; MPV, mean platelet volume; ALT, alanine aminotransferase; AST, aspartate aminotransferase; hpf, high-power field; Auto, automatic analysis

Bloodwork	Reference Range and Units	Patient's Laboratory Value
WBC	4.8-10.8 K/µL	16.3
RBC	4.00-5.20 M/µL	3.80
Hemoglobin	12.0-16.0 g/dL	9.4
Hematocrit	37.0%-47.0%	29.4
MCV	81.0-99.0 fL	77.5
MCH	27.0-31.0 pg	24.8
MCHC	32.0-36.0 g/dL	32.0
RDW	11.5%-14.5%	20.7
MPV	7.2-10.4 fL	8.3
Platelet Count	130-400 K/µL	219
Neutrophil Auto	44.0%-80.0%	87.9
Lymphocyte Auto	13.0%-43.0%	7.4
Monocyte Auto	2.0%-15.0%	4.5
Eosinophil Auto	0.0%-3.0%	0.0
Basophil Auto	0.0%-3.0%	0.2
Absolute Neutrophils	2.1-8.6 K/µL	14.4
ESR	0-25 mm/hour	87
Glucose	74-106 mg/dL	104
Urea Nitrogen	7-17 mg/dL	11
Creatinine	0.5-1.0 mg/dL	0.6
Sodium	137-145 mEq/L	137
Potassium	3.5-5.1 mEq/L	3.6
Chloride	98-107 mEq/L	98
ALT	0-34 U/L	41
AST	14-36 U/L	54
Lactate	0.70-2.00 mmol/L	3.17
C-reactive Protein	<1.0 mg/dL	6.7
Urinalysis (UA)		
Color, UA	Yellow	Light Orange
Specific Gravity	1.001-1.035	1.021
pH	5-9	6.0
Protein, UA	Negative, mg/dL	600
Glucose, Urine	Negative	Negative
Ketones	Negative	Negative
Bilirubin, UA	Negative	Negative
Blood, UA	Negative	Large
Nitrite, UA	Negative	Negative
Urobilinogen	≤1.0 EU/dL	0.2
Leukocytes, UA	Negative	Moderate
WBC, UA	0-5/hpf	10-15
RBC, UA	<4/hpf	25-50
Epithelial Cells	None Seen	Few
Bacteria, UA	None Seen	Moderate
Yeast	None Seen	Moderate
Turbidity	Clear	Cloudy

**Figure 1:**
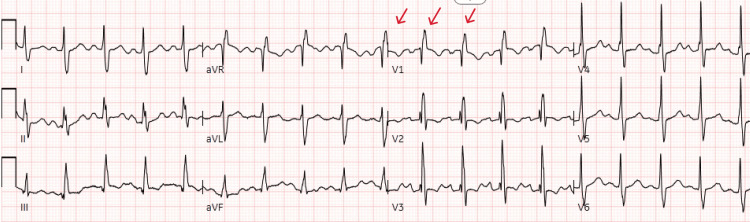
EKG showing sinus tachycardia with HR of 114 beats/minute, right bundle branch block, and right ventricular hypertrophy. EKG, electrocardiogram; HR, heart rate

Electrocardiogram (EKG) showed sinus tachycardia, right bundle branch block, and right ventricular hypertrophy (Figure 1).

**Figure 2:**
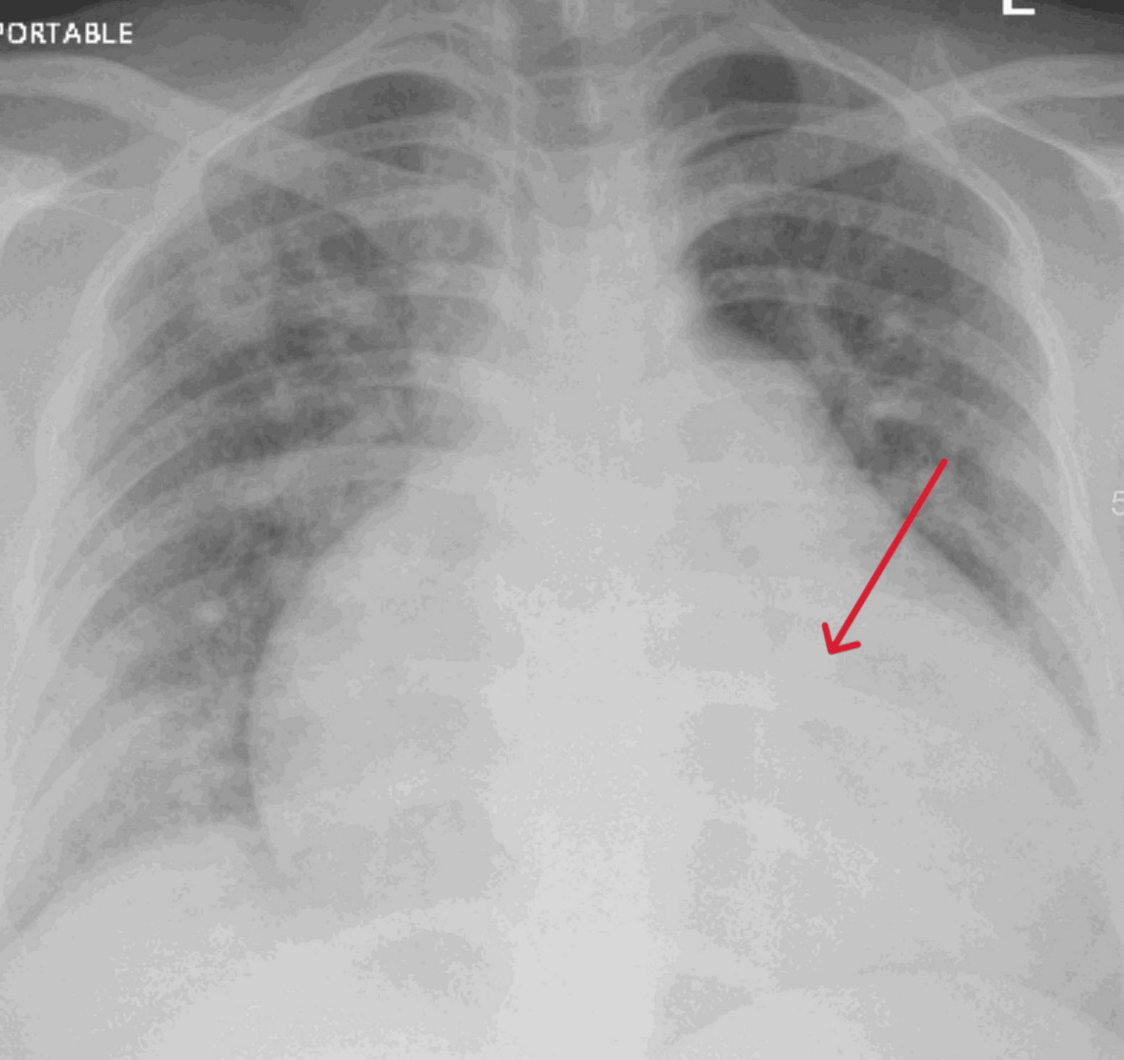
Chest X-ray, AP view, showing enlarged cardiac silhouette with pulmonary vascular congestion.

Chest X-ray demonstrated an enlarged cardiac silhouette with pulmonary vascular congestion (Figure 2).

**Figure 3:**
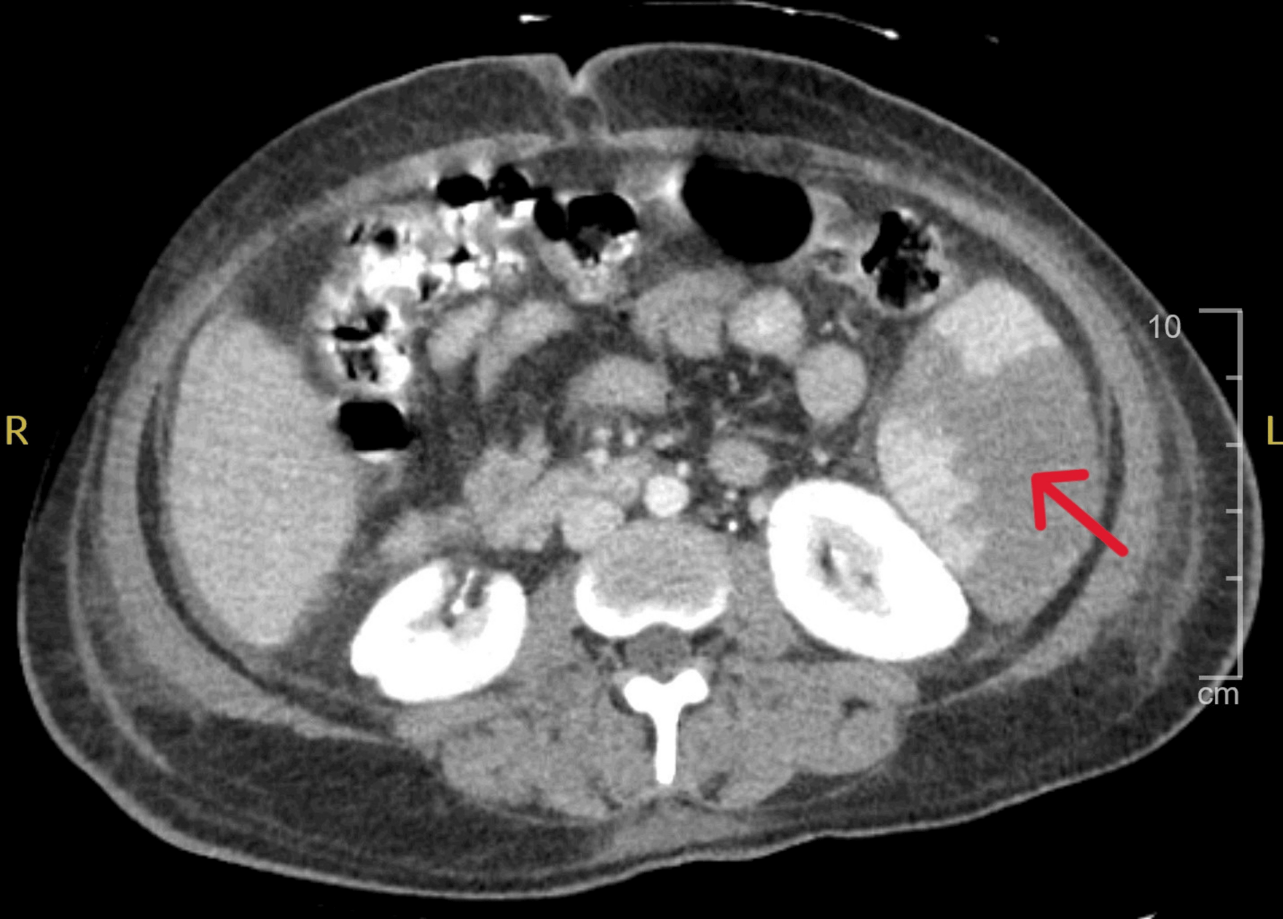
CT of abdomen and pelvis showing splenomegaly with a splenic infarct in the inferior pole. CT: computed tomography

A computed tomography (CT) of the abdomen and pelvis showed liver cirrhosis and splenomegaly with a 15 mm infarct in the inferior pole (Figure 3).

**Figure 4:**
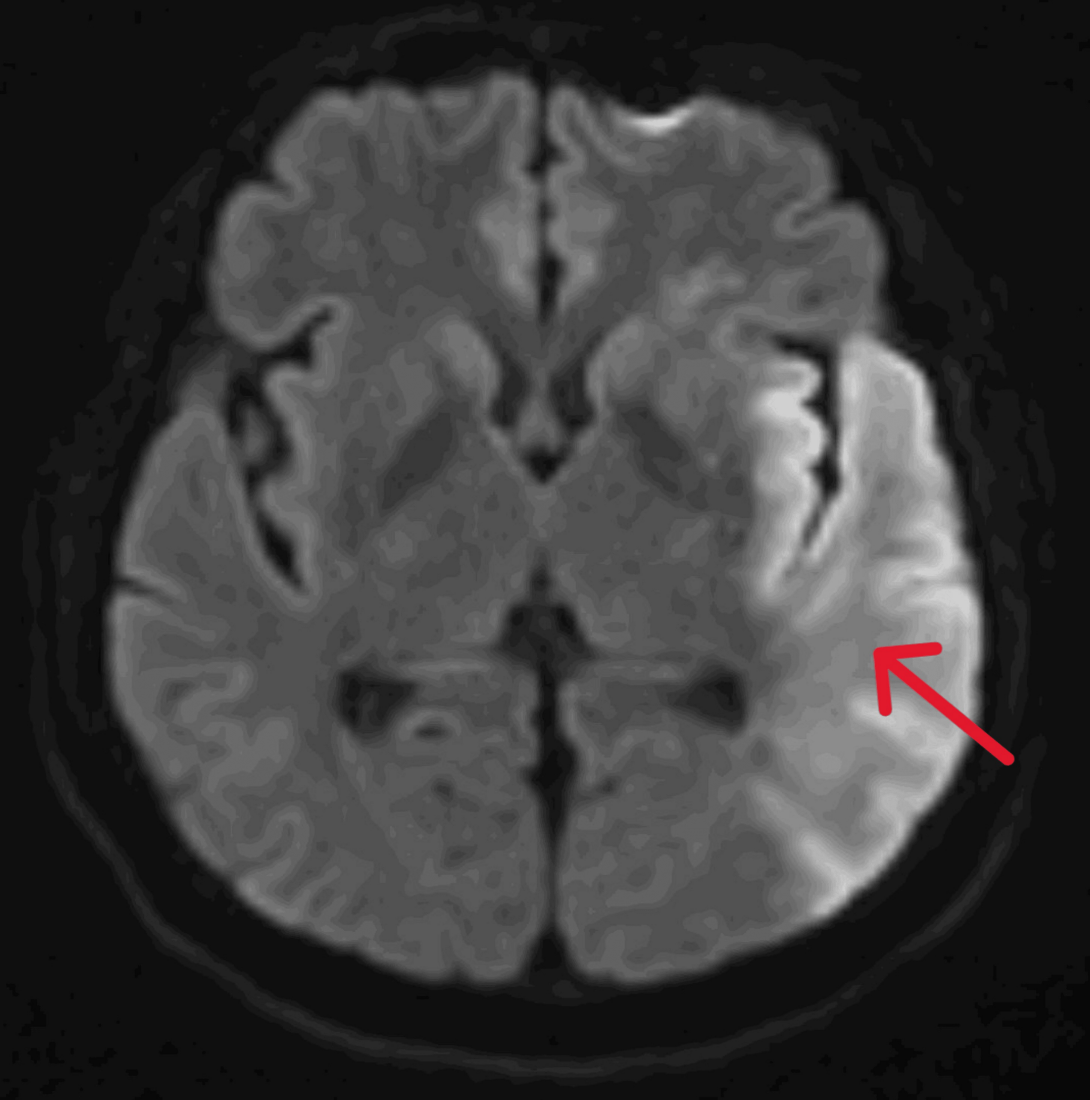
An MRI of the brain showing acute ischemic infarction of the left middle cerebral artery distribution. MRI: magnetic resonance imaging

A CT of the head (CTH) was negative for acute changes. However, a magnetic resonance imaging (MRI) of the brain showed acute ischemic infarction of the left middle cerebral artery distribution (Figure 4). Cerebral CT angiography confirmed a high-grade stenosis of the left middle cerebral artery at the junction of the M1 and M2 segments.

On the repeat neurological examination, no movements were noticed in the right upper and lower extremities (0/5). The patient urgently underwent mechanical thrombectomy with a National Institutes of Health (NIH) score of 20 with final reperfusion with a thrombolysis in cerebral infarction (TICI) score of 3 and an improvement of neurological examination to 4/5 in both right upper and lower extremities and with gradual improvement of mental status. A repeat MRI of the brain was done the next day and showed a hemorrhagic conversion; thus, the patient was on hemicrania watch for five days.

**Figure 5:**
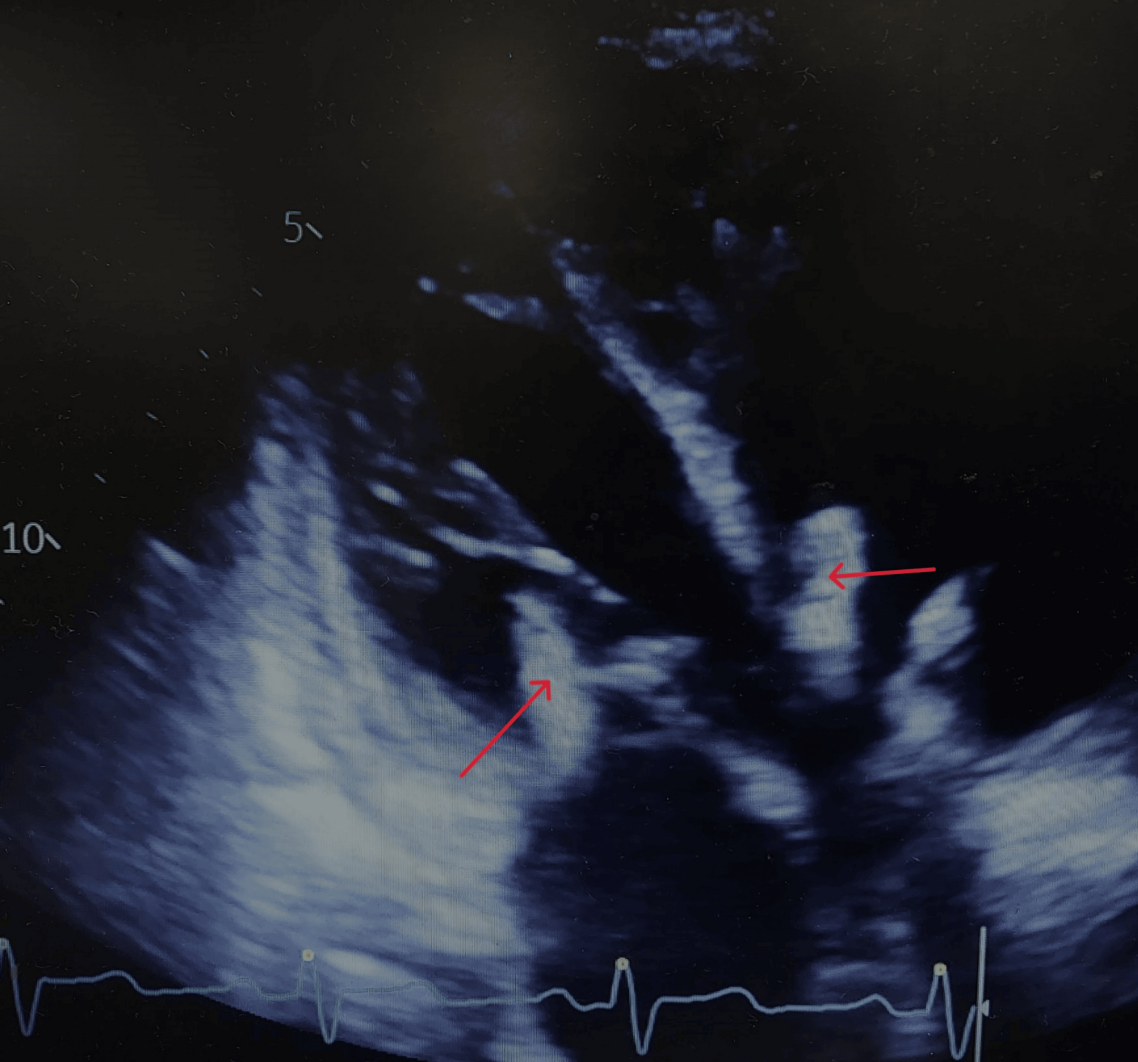
TTE showing the apical four-chamber view, long axis, and left- and right-sided infective endocarditis with mobile vegetations. TTE: transthoracic echocardiography

Transthoracic echocardiography (TTE) was performed and revealed left- and right-sided infective endocarditis with mobile 14 mm vegetation on the mitral valve and 20 mm vegetation on the tricuspid valve (Figure 5 and Video 1). Left ventricular ejection fraction (EF) was decreased to 40%. Also, there was a left-to-right shunt visualized through a patent foramen ovale (PFO) with signs of moderate pulmonary hypertension with a tricuspid pressure gradient of 60 mmHg.

**Video 1:** TTE showing the apical four-chamber view, long axis, and left- and right-sided infective endocarditis with mobile vegetations on the mitral and tricuspid valves. TTE: transthoracic echocardiography

The patient was initially started on broad-spectrum antibiotics vancomycin and cefepime effective against gram-positive bacteria including methicillin-resistant *Staphylococcus aureus*, enterococci, and gram-negative bacteria, including *Pseudomonas aeruginosa*. A blood culture grew *Streptococcus mitis/oralis*, and antibiotics were narrowed to ceftriaxone for a total of six weeks of antibiotics. Repeat CTH on day 3 did not show signs of hemorrhage.

Surgical intervention for infective endocarditis was recommended, and the patient was transferred to the tertiary center for valve replacement surgery. However, at the tertiary center, the patient and the family refused surgery despite understanding the risks and decided to return to their home country.

## Discussion

Our patient demonstrated bilateral MIE involving the mitral and tricuspid valves with large vegetations and systemic complications, including acute ischemic stroke and splenic infarction. The presence of vegetations on both left- and right-sided valves raises suspicion for underlying predisposing factors, such as congenital cardiac anomalies or shunts. In this case, a PFO was identified, which likely facilitated paradoxical embolization. Studies suggest that congenital shunts, such as PFOs, intracardiac devices, or prosthetic valves, can predispose patients to bilateral endocarditis by allowing hematogenous spread between systemic and pulmonary circulation [7-9]. In addition, our patient had poor dentition with caries, which is related to *Streptococcus mitis/oralis* infection. *Streptococcus mitis/oralis*, a viridans group streptococcus, is a causative agent of subacute endocarditis, particularly in patients with underlying structural heart abnormalities or dental manipulation history [10]. While commonly associated with left-sided endocarditis, its role in bilateral MIE is exceedingly rare, underscoring the aggressive nature of this case.

The 14 mm mitral valve vegetation and 20 mm tricuspid valve vegetation observed in this patient represent a high embolic risk, consistent with prior studies that associate vegetations larger than 10 mm with increased rates of systemic and pulmonary embolism [11]. The complications in this case were extensive. Neurological sequelae, such as embolic stroke, occur in 20%-40% of left-sided infective endocarditis cases and are often the first indication of the disease [7,12]. This patient suffered an acute left middle cerebral artery infarction requiring emergent mechanical thrombectomy. Additionally, the splenic infarction and moderate heart failure further complicated the clinical course, both of which are well-documented complications in MIE [9,11].

Surgical intervention is a cornerstone in the management of MIE when complications such as heart failure, large vegetation, or systemic embolism are present. Current AHA/ACC guidelines recommend early surgery for patients with large vegetations (>10 mm) associated with embolic events, severe valvular dysfunction, or heart failure [13]. Given the size of the vegetations, the moderate reduction in left ventricular function (EF: 40%), and systemic embolism, the decision to refer this patient for urgent valve replacement surgery was in accordance with these guidelines. Early surgical intervention has been shown to improve outcomes in cases of complicated infective endocarditis [14].

In addition, there are socioeconomic factors and cultural differences, which may worsen cardiovascular outcomes and significantly impact patient prognosis. These barriers include lower health literacy and delayed care seeking, higher rates of unemployment and a lack of adequate insurance coverage, lower frequency of accepting medical advice and poor medical follow-up leading to reduced access to advanced treatments and higher rates of complications [15]. Addressing disparities is critical to ensuring optimal outcomes for all patients, particularly in severe presentations such as MIE.

Despite advancements in diagnostic tools and treatment strategies, bilateral MIE remains associated with significant morbidity and mortality; thus, early recognition and timely intervention are crucial. A multidisciplinary approach involving cardiology, infectious diseases, neurology, and cardiothoracic surgery is essential for optimizing patient outcomes.

## Conclusions

This case highlights the need for clinicians to maintain a high index of suspicion for MIE in patients presenting with systemic embolic phenomena and underlying heart murmurs, even in immunocompetent patients in the absence of traditional risk factors such as intravenous drug use or prosthetic valves. The unusual bilateral involvement also underscores the role of anatomical anomalies, such as a PFO, in predisposing patients to this rare and aggressive form of endocarditis. Early diagnosis, prompt initiation of antimicrobial therapy, and timely surgical intervention improve clinical outcomes and decrease mortality.
